# Epidemiology of influenza B in Australia: 2001‐2014 influenza seasons

**DOI:** 10.1111/irv.12432

**Published:** 2016-10-14

**Authors:** Aye M. Moa, David J. Muscatello, Robin M. Turner, Chandini R. MacIntyre

**Affiliations:** ^1^ School of Public Health and Community MedicineUniversity of New South WalesSydneyNSWAustralia; ^2^ College of Public Service & Community SolutionsArizona State UniversityPhoenixArizonaUSA

**Keywords:** Australia, epidemiology, influenza B, Victoria lineage, Yamagata lineage

## Abstract

**Background:**

Influenza B is characterised by two antigenic lineages: B/Victoria and B/Yamagata. These lineages circulate together with influenza A during influenza seasons, with varying incidence from year to year and by geographic region.

**Objective:**

To determine the epidemiology of influenza B relative to influenza A in Australia.

**Methods:**

Laboratory‐confirmed influenza notifications between 2001 and 2014 in Australia were obtained from the Australian National Notifiable Diseases Surveillance System.

**Results:**

A total of 278 485 laboratory‐confirmed influenza cases were notified during the study period, comprising influenza A (82.2%), B (17.1%) and ‘other and untyped’ (0.7%). The proportion of notifications that were influenza B was highest in five‐ to nine‐year‐olds (27.5%) and lowest in persons aged 85 years and over (11.5%). Of all B notifications with lineage determined, 77.1% were B/Victoria and 22.9% were B/Yamagata infections. Mismatches between the dominant B lineage in a season and the trivalent vaccine B lineage occurred in over one‐third of seasons during the study years. In general, influenza B notifications peaked later than influenza A notifications.

**Conclusion:**

The proportion of circulating influenza B in Australia during 2001‐2014 was slightly lower than the global average and was dominated by B/Victoria. Compared with influenza A, influenza B infection was more common among older children and young adults and less common in the very elderly. Influenza B lineage mismatch with the trivalent vaccine occurred about one‐third of the time.

## 1 Introduction

Influenza activity is monitored through a complex network of local, regional and national influenza surveillance systems that ultimately feed into the World Health Organization's (WHO) global influenza programme. It is estimated that approximately 3‐5 million cases of severe illness and 250 000‐500 000 deaths occur annually.^1^ Until recent times, influenza A remained the primary focus of influenza control, because of its pandemic potential. In comparison, little was known of the epidemiology and transmission features of influenza B, although epidemics of influenza B have been recognised around the globe for decades.^2^, ^3^, ^4^, ^5^ Influenza B virus was believed to have a slower rate of antigenic evolution than influenza A,^6^ and to cause milder illness than influenza A in the past.^7^ Like influenza A, influenza B has caused localised outbreaks, including in mass gatherings.^2^, ^8^, ^9^, ^10^, ^11^ Moreover, influenza B infection may be underidentified compared with influenza A in healthcare settings,^12^ so the true burden of influenza B may be underestimated.

Influenza B virus circulates in the population alongside influenza A, to varying degrees from season to season and by geographic region.^13^, ^14^ Influenza B was first isolated in the 1940s. The B/Victoria‐like virus strain predominated during the 1980s and the B/Yamagata‐like virus strain emerged in the late 1990s.^15^ These two lineages of influenza B are antigenically and genetically different and studies in ferrets found no cross‐reactivity between the two strains.^16^ In recent decades, both lineages have cocirculated with varying relative intensity in the same season in many parts of the world including Australia.^17^, ^18^

While influenza B is thought to evolve more slowly than A, the interaction between the two virus types played a key role in the viral evolution process.^6^, ^17^ It has been suggested that influenza type B and the influenza A (H1N1) subtype have slower antigenic evolution and fewer epidemic episodes, resulting in fewer global movements, compared with influenza A/H3N2. A complex network of interacting factors such as viral evolution, epidemiology and human behaviour may describe different patterns of global circulation among influenza viruses.^6^, ^19^

It is not clear whether influenza B infection has similar clinical symptoms to influenza A infection including pandemic strain A/H1N1pdm09.^20^ Studies have shown controversial findings of clinical features in cases infected with A and B infections.^21^, ^22^, ^23^ In both paediatric and adult populations, patients with influenza B infection showed similar clinical features compared with influenza A.^22^, ^24^, ^25^, ^26^ Like influenza A, influenza B infection can lead to severe complications and death, in both children and adults.^27^, ^28^, ^29^ However, another study found that abdominal pain, vomiting/diarrhoea, headache, general weakness, rhinorrhoea, pharyngitis and otitis were more common with influenza B infection compared with A/H3N2 in adults.^30^ Pathological evidence of bacterial pneumonia and myocardial injury was reported from the autopsy tissue samples from cases with fatal influenza B infection in both children and adults.^31^ Similar to influenza A, influenza B infection can present with neurological manifestations, such as febrile seizure and encephalitis/encephalopathy in hospitalised children.^28^

Inactivated, trivalent influenza vaccine (TIV) was first introduced in the late 1970s and since then it has been widely used for prevention of influenza infection in many countries.^15^ The trivalent vaccine includes two A strains (A/H1N1, A/H3N2) and one B lineage (either B/Victoria or B/Yamagata). Twice yearly, the WHO carefully reviews circulating influenza strains and recommends strains for inclusion in the TIV for the approaching Northern Hemisphere and Southern Hemisphere influenza season.^32^ Trivalent influenza vaccine only includes one B lineage and thus mismatch with the dominant circulating lineage can occur. Mismatch between the TIV's influenza B lineage and the circulating B lineage occurs at approximately two‐ to four‐year intervals in many countries.^18^ In Australia, partial or complete mismatches of B with circulating lineage have been reported.^33^ It is not fully understood whether vaccination with one B lineage confers significant protection against the other. However, vaccine cross‐reactivity has been reported in adults.^34^, ^35^ Reduced vaccine effectiveness has been observed during seasons in which the vaccine B lineage does not match the circulating B lineage.^36^, ^37^ Since 2012, following recommendations by WHO, a quadrivalent influenza vaccine (QIV) (with two A strains and two B lineages) became available to avoid the risk of B lineage vaccine mismatches. The QIV offers improved protection against unmatched influenza B infections and has comparable safety measures to TIV in both children and adults.^38^, ^39^, ^40^

The incidence of influenza B infection varies considerably by influenza season and by global region.^14^ Here, the aim of our study was to describe the epidemiology and contribution of influenza B relative to influenza A in Australia.

## 2 Methods

We requested weekly, age‐specific counts of laboratory‐confirmed influenza notifications by type, subtype/lineage and age group across all states and territories in Australia between 2001 and 2014, from the National Notifiable Diseases Surveillance System (NNDSS).

In 2001, laboratory‐confirmed influenza infections became notifiable in Australia. A scheduled reporting of laboratory‐confirmed influenza infections is the primary source of influenza data in Australia and influenza notifications are reported initially from laboratories and some practices to regional, state or territory health jurisdictions, which report on to the NNDSS daily. The Australian case definition for notifiable influenza does not specify criteria for testing patients, so selection of patients tested may vary by individual clinician and by regional policy or clinical practice norms. The surveillance case definition for influenza is described below. Only confirmed cases are notified and a confirmed case requires laboratory definitive evidence:^41^

1. Isolation of influenza virus by culture from appropriate respiratory tract specimen, or
2. Detection of influenza virus by nucleic acid testing from appropriate respiratory tract specimen, or
3. Laboratory detection of influenza virus antigen from appropriate respiratory tract specimen, or
4. IgG seroconversion or a significant increase in antibody level or a fourfold or greater rise in titre to influenza virus, or
5. Single high titre by complement fixation test or haemagglutination inhibition assay to influenza virus.

The threshold for a high titre could vary over time and between states and territories.

In addition, a selection of influenza isolates is referred by local laboratories to the WHO Collaborating Centre for Reference and Research on Influenza (WHOCC) which conducts molecular strain typing to inform the WHO for vaccine formulation policy as well as for monitoring genetic changes and antiviral resistance of the influenza viruses. The WHOCC information on circulating strains is reported in national surveillance reports.^42^, ^43^ However, selection of influenza isolates for referral to WHOCC is not nationally standardised and thus may not be a random sample of the case population.

### 2.1 Study data and analysis

In the data set received, the recorded date of onset was used for the disease onset date if available. Otherwise, the earliest of the specimen collection date, or the notification received date was applied. Although there are variations between states and territories, laboratory testing methods used for diagnosis include at least one of the followings: nucleic acid testing, antigen detection, serology and culture. Except earlier years, most of the notifications included influenza type (A or B), but there are no national standards for reporting subtype or lineage, if available, although if the laboratory isolates and notifies a subtype or lineage, it can be recorded in the notification database. Subtyping and lineage determination is undertaken at the discretion of the laboratory and is subject to the tests utilised.

We excluded 18 notifications of the 2009 pandemic influenza strain (influenza A/H1N1pdm09) with an onset date before 2009, due to administrative errors in the data received. A total of 252 notifications with an unknown age (215 notifications for influenza A and 37 notifications for influenza B) were also excluded. We determined the total number and the proportion of laboratory‐confirmed influenza notifications by type in each year for the study period. The proportion of influenza A and B were also estimated by age group and by region (state and territory) for 2001‐2014 seasons. Population data by year and age group were extracted from the Australian Institute of Health and Welfare (AIHW) GRIM books 2013.^44^ We calculated the age‐specific notification rate per 100 000 population for each of influenza A and B for the year, in which influenza B represented at least 20% of notifications in the year. In addition, we examined the proportion of each influenza B lineage across different age groups in the study.

### 2.2 Mismatch with TIV vaccine B lineage

We also determined the number of years where mismatch occurred between the TIV vaccine B lineage and the circulating B lineage in Australia for the study period. The NNDSS also provided subtype and lineage data as part of the data request in our study, but few notifications included the information. Thus, type B lineage mismatch information analysed here was obtained from published influenza surveillance reports.^42^, ^43^, ^45^ Influenza B lineage mismatch was defined as a season when >60% of circulating B lineage virus was different to the lineage included in the TIV for that season. In the season, if both B lineages cocirculated at equal or almost equal proportions (40%‐59%), then a partial mismatch was defined for the year.

In Australia, the influenza season typically begins in May, peaks between July and August and finishes in October, although this can vary.^1^, ^43^ To determine differences in peak seasonal activity between influenza A and B, we determined the week in which the maximum number of notifications was received in the season for each type in each of the 14 years, except 2009. The year 2009 was excluded from this analysis as it was a pandemic year. We then compared the timing of the annual epidemic peak between influenza A and B.

### 2.3 Ethics approval

Ethical approval was received for this study from the University of New South Wales, reference number (HREA—Ref: 2014‐7‐62).

## 3 Results

Overall, there were 278 485 laboratory‐confirmed influenza notifications in Australia during 2001‐2014. Of these, influenza A accounted for 228 990 (82.2%), influenza B for 47 476 (17.1%) and ‘other and untyped’ for 2019 (0.7%). Annual numbers of notifications increased markedly over time. Table 1 shows the percentage of each type of influenza for each year. The proportion of influenza B varied by year, accounting for more than 20% of all notifications in 2002, 2005, 2006, 2008, 2011, 2012 and 2013, and <1% in 2009, the influenza A/H1N1pdm09 pandemic year. Influenza B predominated in only one year, 2008.

**Table 1:** Laboratory‐confirmed influenza notifications by type, Australia, 2001‐2014

Year	Notifications type A (%)	Notifications type B (%)	Notifications Others/Untyped (%)	Total notifications
2001	1005 (77.5)	140 (10.8)	151 (11.7)	1296
2002	2681 (73.3)	868 (23.7)	111 (3.0)	3660
2003	3129 (89.7)	124 (3.6)	237 (6.8)	3490
2004	1574 (76.7)	370 (18.0)	108 (5.3)	2052
2005	3400 (74.4)	1000 (21.9)	169 (3.7)	4569
2006	2343 (70.6)	877 (26.4)	100 (3.0)	3320
2007	9230 (87.3)	956 (9.0)	388 (3.7)	10 574
2008	4023 (43.9)	5029 (54.9)	104 (1.1)	9156
2009	58 411 (99.0)	478 (0.8)	138 (0.2)	59 027
2010	11 976 (89.9)	1282 (9.6)	66 (0.5)	13 324
2011	19 895 (72.6)	7328 (26.8)	170 (0.6)	27 393
2012	33 908 (76.2)	10 538 (23.7)	78 (0.2)	44 524
2013	17 731 (62.8)	10 410 (36.9)	72 (0.3)	28 213
2014	59 684 (87.9)	8076 (11.9)	127 (0.2)	67 887
Total	228 990	47 476	2019	278 485

% represents the proportion of influenza strains within the year.

Source: The National Notifiable Diseases Surveillance System (NNDSS).

**Figure 1:**
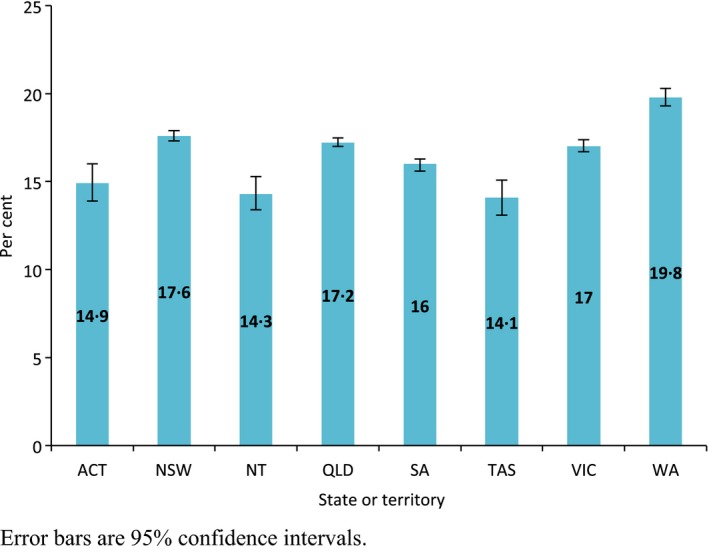
Proportion of influenza notifications that were type B, by state or territory, Australia, 2001‐2014. Error bars are 95% confidence intervals

**Figure 2:**
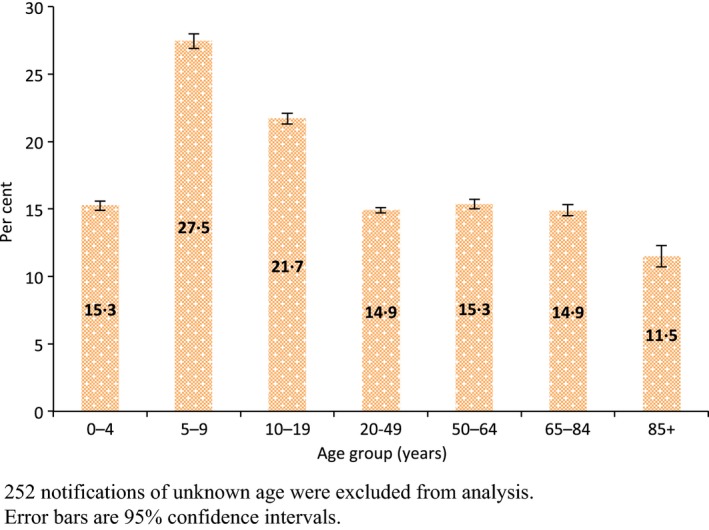
Proportion of influenza notifications that were type B, by age group, Australia, 2001‐2014. 252 notifications of unknown age were excluded from analysis. Error bars are 95% confidence intervals

The proportion of notifications that were type B varied little among Australian states and territories, ranging from 14.1% in Tasmania to 19.8% in Western Australia (Figure 1). Figure 2 shows the proportion of influenza notifications in each age group, which were due to influenza B for the entire study period. The age groups with the highest proportions of influenza B were school‐age children and young adults, with 7639 of 27 808 notifications (27.5%) in five‐ to nine‐year‐olds and 8558 of 39 428 (21.7%) in 10‐ to 19‐year‐olds. The lowest proportion was among persons 85 years and over with 710 of 6174 notifications (11.5%).

**Figure 3:**
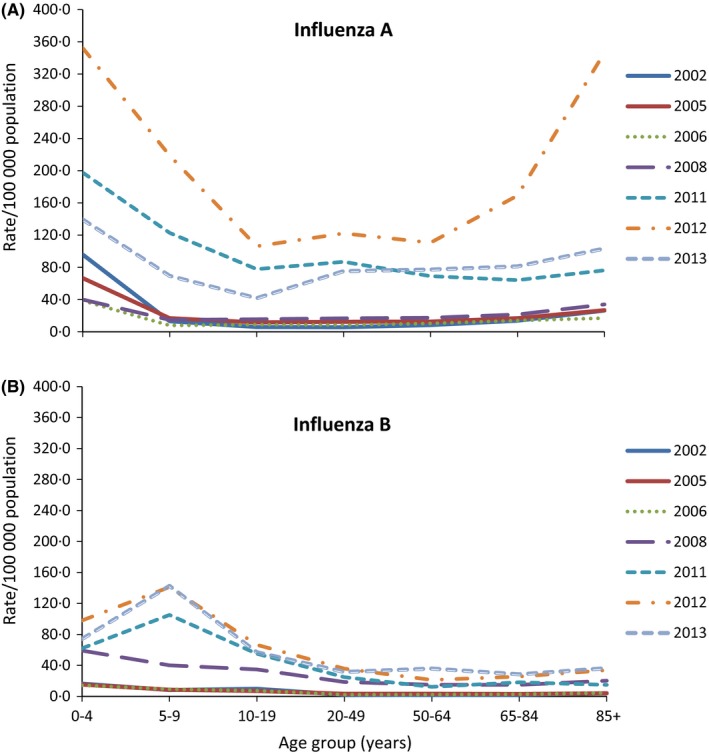
Age‐specific population rate of laboratory‐confirmed influenza A and B notifications in years in which at least 20% of notifications were influenza B, Australia. (A) Top fig: age‐specific notification rate for influenza A (215 notifications of unknown age were excluded from analysis). (B) Bottom fig: age‐specific notification rate for influenza B (37 notifications of unknown age were excluded from analysis)

For seasons in which influenza B represented at least 20% of all notifications, age‐specific notification rates per 100 000 population are shown in Figure 3A,B, for influenza A and B, respectively. Compared with influenza A, higher notification rates of type B were observed in persons aged <20 years and lower rates in persons aged 85 years or more. Compared with other years, 2012 influenza season showed increased influenza activity in the country especially in very young age and the older population.

**Figure 4:**
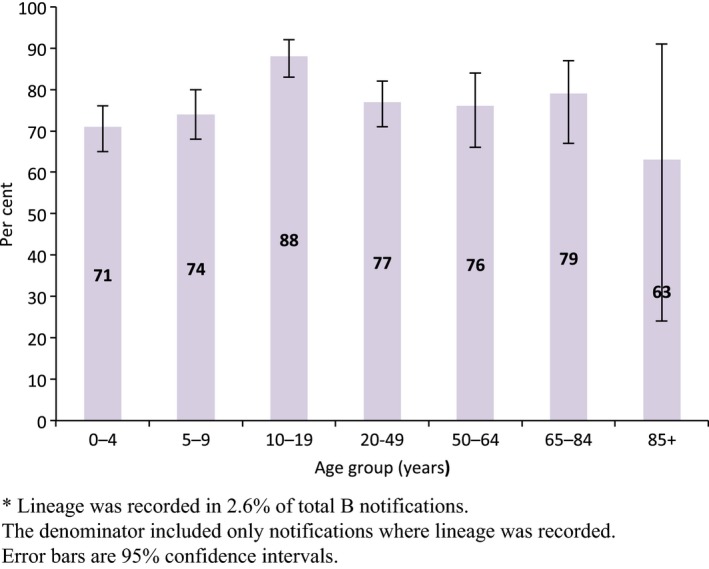
Age‐specific proportions of influenza B notifications that were Victoria lineage, Australia, 2001‐2014*. *Lineage was recorded in 2.6% of total B notifications. The denominator included only notifications where lineage was recorded. Error bars are 95% confidence intervals

Of influenza B notifications, lineage information was reported for 1216 notifications (2.6% of total B cases) over the entire study period. Of these, B/Victoria and B/Yamagata contributed 77.1% and 22.9%, respectively. There was a trend towards a higher proportion of Victoria lineage in 10‐ to 19‐year‐olds. Otherwise, there was limited variation by age (Figure 4). In addition, majority of influenza notifications had limited information regarding further subtypes or lineages in the data set. For influenza B, a small proportion of notifications had lineage information available by year, which is presented in the Table S1.

Table 2 shows the comparison between the circulating B lineage and the vaccine B lineage included in the Southern Hemisphere seasonal TIV in Australia for the study period. Influenza B lineage mismatches occurred in just over one‐third (5 of 14 seasons) as shown in Table 2.

**Table 2:** Circulating influenza B virus lineages in Australia, 2001‐2014 influenza seasons

Year	Dominant circulating B lineage/s	TIV vaccine B lineage	Presence of influenza B lineage mismatch to vaccine
2001	Yamagata	Yamagata	No
2002	Victoria	Yamagata	Yes
2003	Yamagata	Victoria	Yes
2004	Yamagata	Victoria	Yes
2005	Victoria and Yamagata	Yamagata	Partial
2006	Victoria	Victoria	No
2007	Yamagata	Victoria	Yes
2008	Victoria and Yamagata	Yamagata	Partial
2009	Victoria	Yamagata	Yes
2010	Victoria	Victoria	No
2011	Victoria	Victoria	No
2012	Victoria	Victoria	No
2013	Yamagata	Yamagata	No
2014	Yamagata	Yamagata	No

Source: Australian government influenza surveillance reports.^42^, ^43^, ^45^

TIV, Trivalent influenza vaccine; Yamagata = B/Yamagata‐like virus; Victoria = B/Victoria‐like virus.

Mismatch = proportion of circulating B lineage (>60%) being different to the vaccine B strain;

Partial mismatch = proportion of circulating and/or vaccine B lineage in circulation between 40% and 59%.

Timing of peak influenza activity also varied between influenza A and B in the study. The peak influenza B activity appeared later than influenza A in seven seasons (54%), and the epidemic peak of both influenza A and B was overlapped in two seasons [Figures S2 (A,B)]. The difference in peak epidemic activity between influenza A and B was also determined. The peak week of influenza B fell a median 2 weeks later, than that of influenza A over the study period (ignoring pandemic year 2009). The median peak week for influenza A was week 32 (range 32‐39) and for influenza B was week 34 (range 23‐41).

## 4 Discussion

We described the epidemiology and seasonal pattern of influenza B in comparison with influenza A using a national database of laboratory‐confirmed influenza notifications in Australia for 14 influenza seasons. During the study period, both influenza A and B, and each lineage of influenza B cocirculated in all years in varying proportions, and 2008 was the only year in which influenza B predominated over influenza A. Influenza B accounted for 17% of total notifications in the study. This is slightly lower than the global proportion, with an average of 20% estimated over a similar period from 26 countries across different geographic regions including Australia.^14^ In that study, the highest median proportion of influenza B was found in tropical regions and the lowest was in the Southern Hemisphere, but when compared between the geographic regions, the differences were not statistically significant. An explanation for the lower proportion in Australia may arise from the higher incidence of influenza B in younger age groups, combined with Australia's older age distribution compared with the aggregated global population.^46^ In addition, our study showed a broadly similar proportion of influenza B infection across Australia. As influenza typing to the A and B level was virtually complete in our data, we believe that this result is not geographically biased.

Consistent with other studies,^14^, ^47^, ^48^ influenza B affected children and teenagers more than adults in the study. We found that 2012 had a higher rate of influenza A notifications, with increased notifications particularly in younger children and older adults. This was likely due to higher circulation of A/H3N2 during that season and changes in the viral subtype after the sustained presence of the pandemic 2009 strain for the preceding years.^49^ In Australia, 2012 was reported as a severe influenza season, as A/H3N2 tends to be, with increased influenza‐related morbidity and mortality observed.^50^, ^51^

In general, peak influenza B activity arrived later in the season than peak influenza A, and both types cocirculated in each season to varying amounts. However, this relative incidence pattern can vary across different regions of the world,^52^ although our finding is comparable to results from France, where influenza B peaked an average of 3.8 weeks later than influenza A.^53^

Mismatch between the B lineage included in the TIV and the circulating B lineage was observed in just over one‐third of the 14 Australian seasons studied. This result is lower than reported in the United States (46%, from 2001/2002 to 2010/2011 seasons) and the Europe (58%, between 2003/2004 and 2010/2011 seasons).^18^ Again, this varies by regions and influenza season globally.^14^, ^18^

In our study, lineage was reported in 2.6% of the total B notifications, and B/Victoria predominated B/Yamagata throughout the entire study period. Nevertheless, both lineages cocirculated each year. Data from other countries and from another Australian study found that B/Victoria cases were younger than cases with B/Yamagata infection.^33^, ^54^, ^55^ Our results showed the only age group with a trend towards a higher proportion of Victoria lineage was 10‐ to 19‐year‐olds. Although the actual reasons were not fully understood, one study proposed that the haemagglutinin antigen to host receptor binding preferences vary between the two lineages according to the infected person's age.^33^

Although influenza type B constitutes a smaller proportion of influenza burden than type A during seasonal epidemics, a significant impact of influenza B has been reported in all age groups globally.^56^, ^57^ A French population‐based disease burden study estimated that influenza B resulted in an estimated total cost of 145 million Euros (95% CI: 88‐201 million Euros) to the French Health Insurance system in the 2010‐2011 influenza season.^58^ Cost per case was highest in working‐aged adults when considering both direct and indirect costs such as work absenteeism, but this finding may vary according to a country's health insurance system.

At present, QIV has limited production capacity, and vaccine manufacturers have great interest in manufacturing and licensing of QIV due to the potential benefits of an additional B lineage in the vaccine. Many studies have reported the cost‐effectiveness and advantages of QIV over the trivalent vaccine.^59^, ^60^, ^61^, ^62^, ^63^ If TIVs were replaced with QIVs in immunisation programmes; this could provide benefit at both the individual and population levels through reduced numbers of infections arising from TIV influenza B and circulating influenza B lineage mismatch. However, adoption of QIV may be constrained in some settings depending on the resources and healthcare system capacity in the country. Thus, switching to QIV should be considered according to available resources, with emphasis on high‐risk groups. In addition, there needs to be more research into the effectiveness and cost‐benefit profile of QIV over TIV.

There are limitations in the study. Our study included data only from notified cases, and thus, laboratory‐confirmed influenza notifications may underestimate the true incidence of influenza infections in the population.^12^ Only a small proportion of persons infected with influenza will seek medical attention and therefore have the opportunity to be tested. Testing of patients seeking medical care is at the discretion of the medical practitioner, or may vary by local testing conventions, and these practices may vary over time. Besides, there were increased notifications received by NNDSS in the later years during the study; therefore, it is important to interpret these changes over time with caution. We noticed that the total number of influenza notifications were high in 2009, a pandemic year which triggered a much higher demand for testing, and from 2011 onwards. However, the reasons for increased testing/notifications are not fully understood.^64^, ^65^ Moreover, diagnostic practices and test types used by laboratories may change over time, and by laboratory (public and private) and by regional health jurisdiction, especially in relation to subtyping or genotyping of influenza viruses. However, these biases are offset in our study because we focused mainly on differences between influenza types A and B and between lineages of influenza B, and clinicians cannot know what influenza strain the patient has when deciding to take a specimen. Again, conventions or policies for testing of samples may vary by age group and other motivating factors. For example, more testing was carried out in children than in adults during the pandemic period (2009) compared with other years, and more testing was done overall in paediatric population than adults.^65^ Lastly, due to the small proportions of influenza B with lineage information available and the lack of standardised strain typing practices by laboratories across Australia, these results may not necessarily be representative of all influenza B notifications. Nevertheless, laboratory‐confirmed influenza notifications received nationally provide timely and very specific information on circulating influenza viruses to inform influenza prevention and control programmes in the country.

## 5 Conclusion

Our findings indicated that proportion of influenza B circulating in Australia is slightly lower than the global average of one‐fifth of circulating influenza types. The proportion of influenza B varied over the 14 epidemic seasons, and influenza B predominated in only one year (2008) during the study period. Seasonal mismatches between the circulating B lineage and the B lineage in the TIV occurred in just over one‐third of seasons. The use of quadrivalent vaccines could provide potential benefits in preventing the burden of influenza B.

## Conflict of Interests

AMM, DJM, RMT: Nothing to declare. CRM: CRM has received in‐kind support and funding for investigator‐driven research from GlaxoSmithKline, Pfizer, Merck, and bioCSL, and has sat on advisory boards for Merck, GlaxoSmithKline and Pfizer.
