# Supplementary figures and images for: Epidemiology of influenza B in Australia: 2001‐2014 influenza seasons
Source: Influenza Other Respir Viruses. 2016 Oct 14;11(2):102–9. doi: 10.1111/irv.12432 (PMC5304570; doi:10.1111/irv.12432)

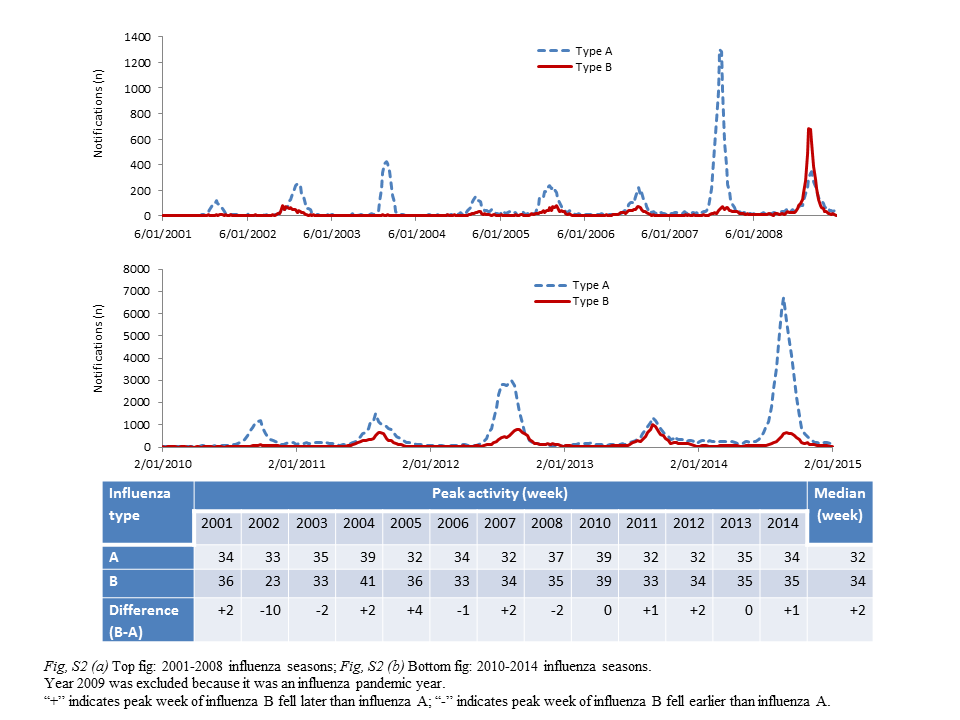

Supplement: Supplementary file 1 [file IRV-11-102-s001.tif]
