# Supplementary material for: Epidemiology of influenza B in Australia: 2001‐2014 influenza seasons
Source: Influenza Other Respir Viruses. 2016 Oct 14;11(2):102–9. doi: 10.1111/irv.12432 (PMC5304570; doi:10.1111/irv.12432)
Supplement: Supplementary file 2 [file IRV-11-102-s002.docx]

**Supporting information:**

S1: Total notifications of influenza B and B lineages by year, Australia, 2001-2014.

| Year | B/Victoria lineage (%) | B/Yamagata lineage (%) | B/unknown (%) | Total number of influenza B notifications (N) |
| --- | --- | --- | --- | --- |
| 2001 | 0 (0.0) | 0 (0.0) | 140 (100) | 140 |
| 2002 | 0 (0.0) | 0 (0.0) | 868 (100) | 868 |
| 2003 | 1 (0.8) | 0 (0.0) | 123 (99.2) | 124 |
| 2004 | 0 (0.0) | 2 (0.5) | 368 (99.5) | 370 |
| 2005 | 0 (0.0) | 1 (0.1) | 999 (99.9) | 1,000 |
| 2006 | 2 (0.2) | 0 (0.0) | 875 (99.8) | 877 |
| 2007 | 2 (0.2) | 14 (1.5) | 940 (98.3) | 956 |
| 2008 | 154 (3.1) | 236 (4.7) | 4,639 (92.2) | 5,029 |
| 2009 | 0 (0.0) | 0 (0.0) | 478 (100) | 478 |
| 2010 | 49 (3.8) | 0 (0.0) | 1,233 (96.2) | 1,282 |
| 2011 | 186 (2.5) | 4 (0.1) | 7,138 (97.4) | 7,328 |
| 2012 | 258 (2.4) | 19 (0.2) | 10,261 (97.4) | 10,538 |
| 2013 | 197 (1.9) | 3 (0.0) | 10,210 (98.1) | 10,410 |
| 2014 | 88 (1.1) | 0 (0.0) | 7,988 (98.9) | 8,076 |
| Total | 937 | 279 | 46,260 | 47,476 |

Source: The National Notifiable Diseases Surveillance System (NNDSS)
